# Bisphenol A Causes Liver Damage and Selectively Alters the Neurochemical Coding of Intrahepatic Parasympathetic Nerves in Juvenile Porcine Models under Physiological Conditions

**DOI:** 10.3390/ijms18122726

**Published:** 2017-12-15

**Authors:** Michael Thoene, Liliana Rytel, Ewa Dzika, Andrzej Włodarczyk, Ewa Kruminis-Kaszkiel, Ptaszyński Konrad, Joanna Wojtkiewicz

**Affiliations:** 1 Department of Medical Biology, Faculty of Health Sciences, University of Warmia and Mazury, 10-561 Olsztyn, Poland; e.dzika@uwm.edu.pl; 2 Department of Internal Medicine and Clinic, Faculty of Veterinary Medicine, University of Warmia and Mazury, 10-718 Olsztyn, Poland; Liliana.Rytel@uwm.edu.pl; 3 Department of Public Health, Epidemiology and Microbiology, School of Medicine, University of Warmia and Mazury, 10-718 Olsztyn, Poland; andrzej.wlodarczyk@uwm.edu.pl; 4 Department of Pathophysiology, School of Medicine, University of Warmia and Mazury, 10-718 Olsztyn, Poland; ewa.kruminis@uwm.edu.pl (E.K.-K.); joanna.wojtkiewicz@uwm.edu.pl (J.W.); 5 Department of Pathomorphology, School of Medicine, University of Warmia and Mazury, 10-718 Olsztyn, Poland; konrad.ptaszynski@gmail.com; 6 Laboratory of Regenerative Medicine, University of Warmia and Mazury in Olsztyn, 10-900 Olsztyn, Poland

**Keywords:** bisphenol A, hepatic toxicity, immunofluorescence technique, parasympathetic nervous system, metabolic disorders

## Abstract

Bisphenol A (BPA) is an extremely common polymer that is used in typical everyday products throughout the world, especially in food and beverage containers. Within the last ten years, it has been found that the BPA monomer tends to leach into foodstuffs, and nanogram concentrations of it may cause a variety of deleterious health effects. These health problems are very evident in developing children and in young adults. The aim of this study was to expose developing pigs to dietary BPA at both legally acceptable and ten-fold higher levels. Livers that had been exposed to BPA showed vacuolar degeneration, sinusoidal dilatation, vascular congestion and glycogen depletion that increased with exposure levels. Furthermore, the livers of these models were then examined for irregularities and double-labeled immunofluorescence was used to check the innervated hepatic samples for varying neuronal expression of selected neuronal markers in the parasympathetic nervous system (PSNS). It was found that both the PSNS and all of the neuronal markers showed increased expression, with some of them being significant even at recommended safe exposure levels. The implications are quite serious since these effects have been observed at recommended safe levels with expression increasing in-line with exposure levels. The increased neuronal markers studied here have been previously correlated with behavioral/psychological disorders of children and young adults, as well as with childhood obesity and diabetes. However, further research must be performed in order to develop a mechanism for the above-mentioned correlations.

## 1. Introduction

Currently, bisphenol A (BPA) can be found throughout our environment. It is a chemical compound commonly used as an epoxy resin in typical everyday products, such as the lining inside canned foods and beverages, pre-packed foodstuffs, packaged baby formula, baby bottles, and containers used for food-storage in the home. Also, it may be found in dental prosthetics, and sales receipts that use thermal paper [1,2]. Unfortunately, some of this resin may leak from the polymer and enter into the human body, mainly through the contamination of food and beverages. The United States Environmental Protection Agency (EPA) has declared that BPA is the third highest priority environmental hazard [3,4]. BPA has been used for many years, because it was originally thought to be a safe chemical that showed very little chemical toxicity during standard testing. However, bisphenol is not a typical toxin. Instead, it is an EDC (endocrine disrupting compound), and behaves like estrogen in vivo. Therefore, BPA is blocking and/or interfering with the normal functioning of estrogen within the human body. It has been implicated in the disruption of thyroid hormone receptors, androgen receptors, and other endocrine system signaling pathways [5]. Meanwhile, many studies have shown that nanogram levels of bisphenol may be significantly affecting biological systems because of the disruption of normal hormonal signaling pathways. The main clinical manifestations that have been associated with BPA in the literature include reproductive disorders in adults, psychological and metabolic disorders in children, and neoplasms resulting from a weakening of the immune system [5,6,7].

There have been a handful of studies that have associated BPA exposure with abnormal brain development, but usually with limited mechanistic details. There have been no studies (to the best of our knowledge) concerning BPA exposure and the development of innervation in hepatic tissue. Unfortunately, bisphenol exposure is such a new area of research that most of the mechanisms involved have yet to be described.

However, there are a few key studies that have begun to bring these mechanisms to light. Hajszan and Leranth found that “BPA completely negates the ∼70–100% increase in the number of hippocampal and prefrontal spine synapses induced by both estrogens and androgens” [8].

Synaptic loss of this magnitude may have significant consequences, potentially causing cognitive decline, depression, and schizophrenia, according to the authors [8]. These studies were mainly performed on primates. Unfortunately, a molecular basis for these findings has not yet been determined. A study of zebra fish noted that very low-dose bisphenol exposure caused significant abnormal neurogenesis of the hypothalamus which is “a highly conserved brain region involved in hyperactivity” [9]. Again, the molecular mechanisms for this are not clear. Further research using rodent models has attempted to make some of the molecular mechanisms clearer [10,11], but the researchers could only conclude that BPA exposure changes the developing brain, but the mechanisms are still unknown. In terms of a mechanism for how bisphenol is changing neuronal architecture in the brain, and perhaps in the peripheral nervous system as well, one study has found that BPA may cause neuronal changes by inhibiting the proper functioning of T-type calcium channels. The authors have suggested that BPA may act as a modifier of channel gating and may directly plug conductive channel pores [12]. Other authors have found that the biologically active from of bisphenol can bind directly to DNA [13]. However, a mechanism for how that binding would affect neuronal expression is not yet available.

Despite a reasonable amount of recent literature concerning the effects of BPA on the central nervous system (CNS), there are scant articles concerning the effects of BPA on the PNS (peripheral nervous system). Articles concerning the effects of BPA on hepatic tissue are limited to pathophysiological analyses of hepatic tissue at recommended safe levels [14,15]. Some studies have gone a bit farther and have developed a few mechanisms for the hepatic injury. For example, one study found that rats after moderately long-term exposure had hepatocyte damage that was mediated by mitochondrial apoptosis [16]. Another study found that BPA induced DNA damage in human hepatocytes in vitro and in rat hepatocytes in vivo [17]. Unfortunately, there are no articles to date that have examined the pattern of innervation in the nerve fibers of hepatic tissue after BPA exposure, to the best of our knowledge.

The aim of this study was to examine the effects of low and high doses of bisphenol A on the innervation of porcine hepatic tissue, namely, the parasympathetic nervous system (PSNS). Co-localization of vesicular acetylcholine transporter (VAChT), a specific marker for the PSNS [18,19] with cocaine and amphetamine regulated transcript (CART), galanin (GAL), calcitonin gene regulated peptide (CGRP), substance P (SP), and pituitary adenylate cyclase activating polypeptide (PACAP), was studied using standard double-immunofluorescence.

The choice of using the domestic pig for this experiment is not accidental. Due to neurochemical similarities in the organization of the nervous system between human and pig [20,21,22], this mammal species seems to be a good animal model of the processes connected with the influence of various pathological stimuli on the human nervous system. So, the results obtained during the present study may be an animal model of BPA, but they have similarities to the innervation of human liver.

## 2. Results

### 2.1. Histopathological Examination and Biochemical Blood Study

**Figure 1:**
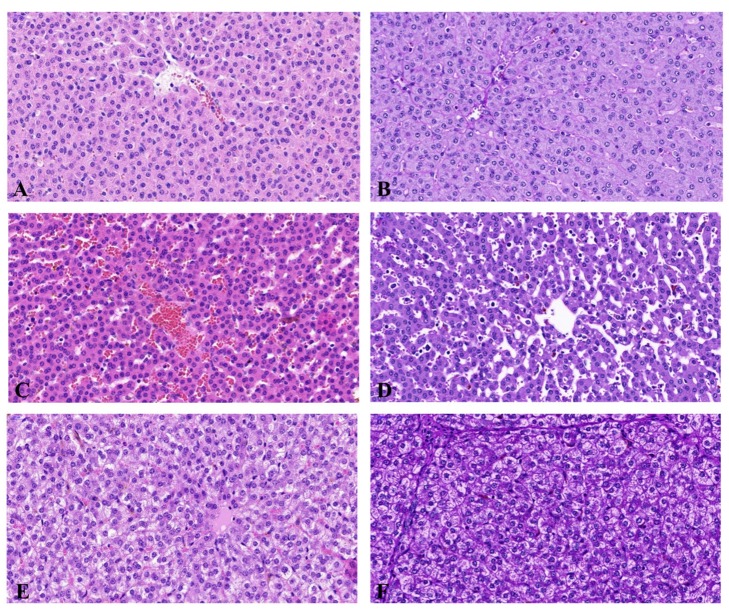
Histopathologic examination of the liver. (**A**,**B**) Liver sections of the control group show unremarkable liver histopathology. Periodic acid-Schiff (PAS) stain exhibits a positive stain of glycogen in the cytoplasm; (**C**,**D**) Liver sections of the low dose bisphenol A (BPA) group show dilated and congested central veins and hepatic sinusoids. PAS stain is normal with a positive cytoplasmic stain; (**E**,**F**) Liver sections of the high dose BPA experimental group show hepatocytes with marked vacuolar degeneration, PAS stain shows changes consistent with glycogen depletion; (**A**,**C**,**E**) 400×; H&E stain; (**B**,**D**,**F**) 400×; PAS stain.

The histological appearance of porcine liver subjected to 0.05 mg/kg bw/day of BPA was characterized as having central vein and hepatic sinusoid dilatation, as well as vascular congestion. Moreover, those samples subjected to 0.5 mg/kg bw/day BPA showed remarkable vacuolar degeneration. All other components of the liver, such as the lobules, appeared normal. The histopathology of the porcine liver control samples displayed unremarkable liver lobular structure with normal hepatocytes and sinusoids. Liver sections of the high dose BPA experimental group showed hepatocytes with marked vacuolar degeneration, and staining showed changes consistent with glycogen depletion. Representative images of the histopathological samples are shown in Figure 1.

Moreover, during the present study, significant changes in the levels of serum hepatic biomarkers (alanine aminotransferase (ALT) and aspartate aminotransferase (AST)) between the control group and both experimental groups were observed. Under physiological conditions, the concentration of ALT amounted to 43 ± 2.4 U/L, and that of AST was 48 ± 5.8 U/L. Low dose BPA caused an increase in the levels of these biomarkers to 69.8 ± 0.8 U/L (ALT) and 66.8 ± 3.4 U/L (AST). In animals that received high doses of BPA, the levels of ALT and AST were even higher and achieved 83.2 ± 3.5 and 87.2 ± 4.1 U/L, respectively.

### 2.2. Neurochemical Characterization of Intrahepatic Nerves

Both doses of BPA studied during the present investigation caused an increase in the number of VAChT-positive nerves (Table 1), but the observed changes were not statistically significant. At suggested safe levels, the number of VAChT^+^ fibers was elevated by 8.8%, and was elevated by 27.0% at a BPA exposure ten times higher than the recommended safe level when compared to the control samples.

At suggested safe-levels, the number of CART^+^/VAChT^+^ fibers was 45.3% higher than that of the control samples, but was not statistically significant. At an exposure of ten times higher than recommended safe levels, the density of CART^+^/VAChT^+^ nerves was 190.6% higher than that of the control samples, and was statistically significant. Also, at suggested safe-levels, the number of nerves simultaneously immunoreactive to SP and VAChT was 41.9% higher than that of the control samples, and at an exposure of ten times higher than recommended safe levels, the density of SP^+^/VAChT^+^ fibers was 43.5% higher than that of the control samples. However, neither result was statistically significant.

**Figure 2:**
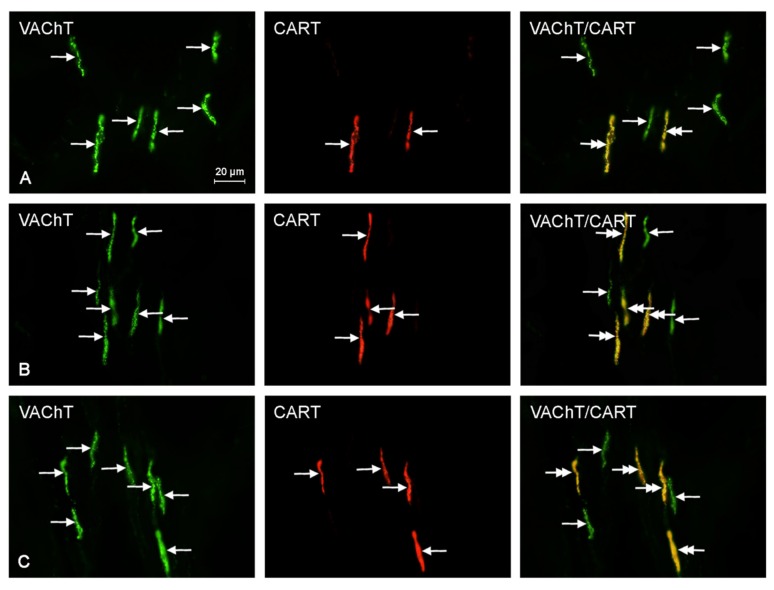
Representative images of immunocytochemical localization of VAChT-, and CART-immunoreactive nerve fibers in intrahepatic sympathetic nerves (arrows) of the control (CTRL; (**A**)), E1—experimental group 1 (low dose of bisphenol A; (**B**)), and E2 – experimental group 2 (high dose of bisphenol A, (**C**)); VAChT or CART-IR nerve fibers are indicated with arrows, VAChT and CART-IR nerve fibers are indicated with double-headed arrows; scale bars in all figures; 20 μm.

**Figure 3:**
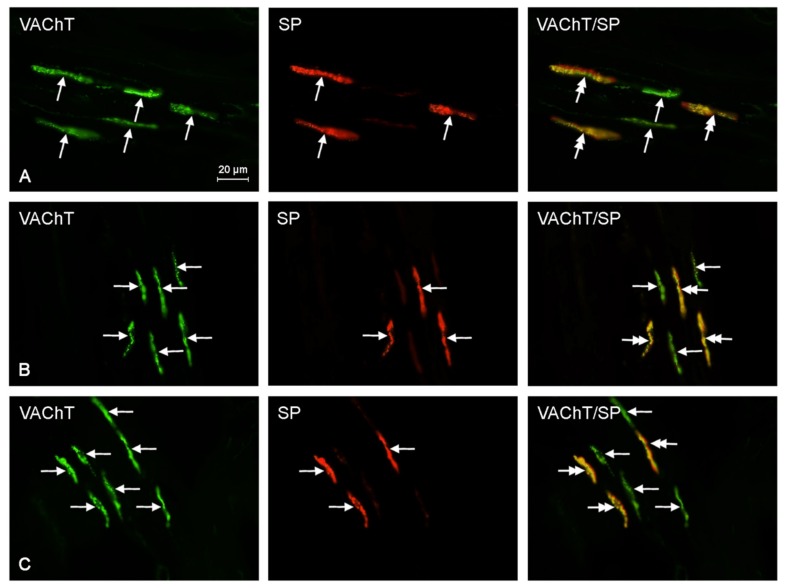
Representative images of immunocytochemical localization of VAChT- and SP-immunoreactive nerve fibers in intrahepatic sympathetic nerves (arrows) of the control (CTRL; (**A**)), E1—experimental group 1 (low dose of bisphenol A; (**B**)), and E2—experimental group 2 (high dose of bisphenol A, (**C**)); VAChT or SP-IR nerve fibers are indicated with arrows, VAChT and SP-IR nerve fibers are indicated with double-headed arrows; scale bars in all figures; 20 μm.

**Figure 4:**
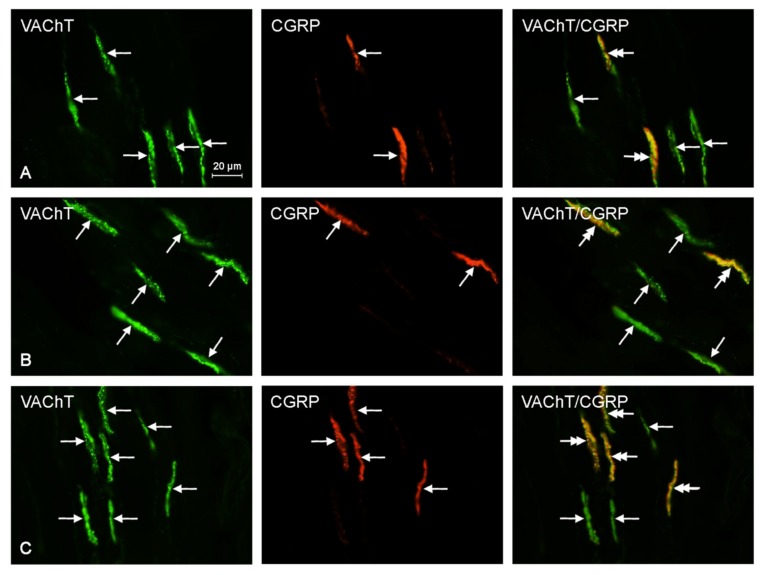
Representative images of immunocytochemical localization of VAChT- and CGRP-immunoreactive nerve fibers in intrahepatic sympathetic nerves (arrows) of the control (CTRL; (**A**)), E1—experimental group 1 (low dose of bisphenol A; (**B**)), and E2—experimental group 2 (high dose of bisphenol A, (**C**)); VAChT or CGRP-IR nerve fibers are indicated with arrows, VAChT and CGRP-IR nerve fibers are indicated with double-headed arrows; scale bars in all figures; 20 μm.

**Figure 5:**
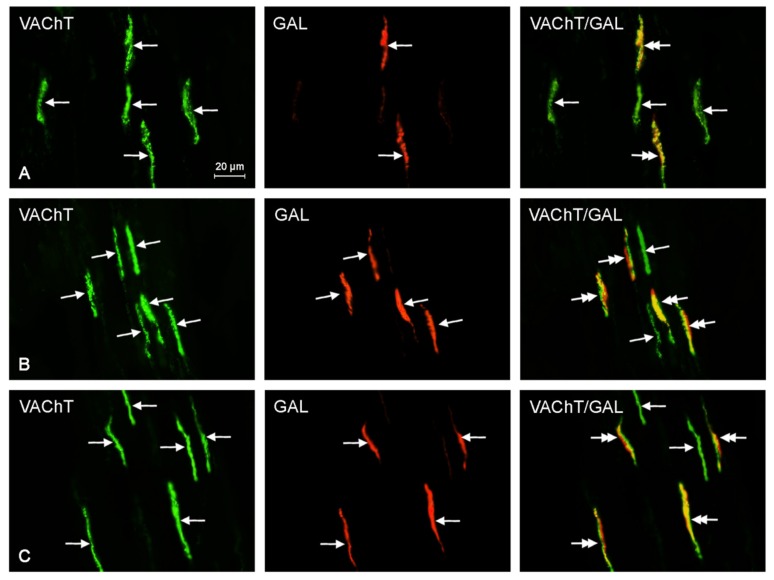
Representative images of immunocytochemical localization of VAChT- and GAL-immunoreactive nerve fibers in intrahepatic sympathetic nerves (arrows) of the control (CTRL; (**A**)), E1—experimental group 1 (low dose of bisphenol A; (**B**)), and E2—experimental group 2 (high dose of bisphenol A, (**C**)); VAChT or GAL-IR nerve fibers are indicated with arrows, VAChT and GAL-IR nerve fibers are indicated with double-headed arrows; scale bars in all figures; 20 μm.

**Figure 6:**
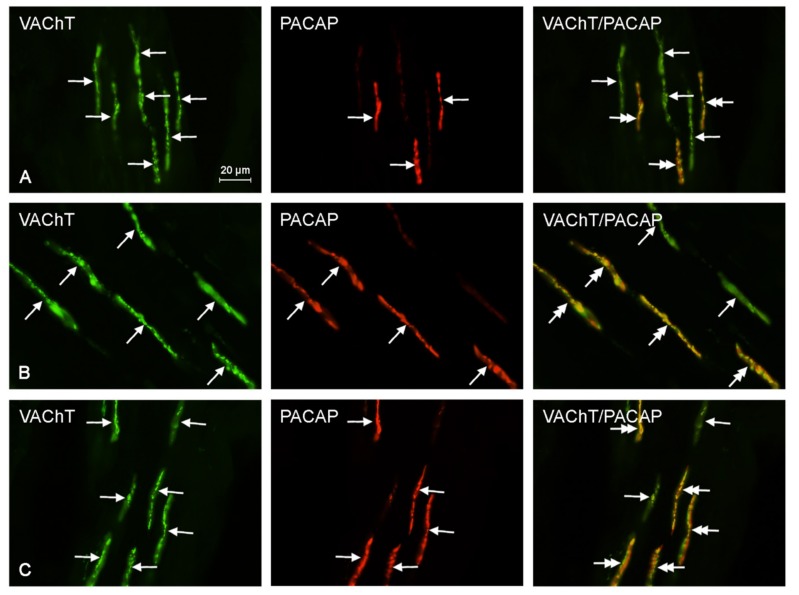
Representative images of immunocytochemical localization of VAChT- and PACAP-immunoreactive nerve fibers in intrahepatic sympathetic nerves (arrows) of the control (CTRL; (**A**)), E1—experimental group 1 (low dose of bisphenol A; (**B**)), and E2—experimental group 2 (high dose of bisphenol A, (**C**)); VAChT or PACAP-IR nerve fibers are indicated with arrows, VAChT and PACAP-IR nerve fibers are indicated with double-headed arrows; scale bars in all figures; 20 μm.

There was an increase of 13.3% in the percentage of CGRP^+^/VAChT^+^ fibers at recommended safe levels, but it was not statistically significant. At ten times the legally recommended safe level, there was a statistically significant increase of 70.0% as compared to that of the control samples. Furthermore, at the suggested safe-level, the number of GAL^+^/VAChT^+^ nerves was 167.6% higher than that of the control samples, and at an exposure of ten times higher than recommended safe levels, the expression of nerves immunoreactive to GAL and VAChT was 258.8% higher than that of the control sample. Both of these results were statistically significant. Finally, the number of PACAP^+^/VAChT^+^ nerves at suggested safe-levels was 49.2% higher than that of the control samples, and at an exposure of ten times higher than recommended safe levels, the expression of PACAP^+^/VAChT^+^ nerves was 64.4% higher than that of the control samples. Unfortunately, these results were not statistically significant. The results above are tabulated in Table 1, and representative images of the immunocytochemical co-localization of VAChT with the chosen neuronal markers are shown in Figure 2, Figure 3, Figure 4, Figure 5 and Figure 6.

## 3. Discussion

There are several articles dealing with hepatic damage. Quite often, the damage takes the form of central vein and hepatic sinusoid dilatation, vascular congestion, and vacuolar degeneration [23,24]. These particular histopathologies are typical of hepatic changes commonly observed after chemical exposure, with much of the literature centering on the effects of long-term ethanol or narcotics usage [25,26]. However, there are several toxins other than ethanol or narcotics that cause hepatic damage. Since, ethanol and narcotics are most often intentionally abused; they tend to get more attention in the scientific literature. However, there are other chemicals that may cause similar changes to the hepatic tissue. For example, acetaminophen (paracetamol) is a common over the counter pain reliever, which may cause extensive hepatic trauma even after only a single over-exposure [27]. Environmental exposure to industrial toxins such as carbon tetrachloride (CCl4) has also been shown to cause vascular congestion and vacuolar degeneration [28,29]. Exposure of endogenous wildlife to a popular insecticide also caused observable hepatic damage in a similar way to that of CCl4 [30]. Furthermore, life-saving drugs such as the antiretroviral HIV treatment azidothymidine (AZT) and popular anti-cancer chemotherapy medications have been shown to cause similar hepatic trauma [31,32]. What all of these potentially hepatotoxic compounds have in common is vascular congestion, vacuolar degeneration, and quite often oversized mitochondria, which is a sign of mitochondrial damage [25]. Very often, this hepatic damage is not only observed in microscopic tissue samples, but is also recorded in specialized markers found in the blood serum.

The types of hepatic injury mentioned above are very often measurable with the use of serum alanine aminotransferase (ALT) as well as aspartate aminotransferase (AST) levels. ALT and AST are transaminases widely used to assess hepatic trauma. AST is not a specific hepatic marker since it is often released when other cellular damage occurs, for example, during a heart lesion, over-exertion of skeletal muscle, or some erythrocyte based diseases [27]. ALT is far more specific than AST in detecting damaged hepatocytes, and also remains in the serum for longer. However, both are normally reported together in the literature, and increased levels are considered to be an indicator of hepatic damage.

One of the main ways that hepatocytes are damaged is via mitochondrial enlargement and vacuolar degeneration, which are both caused directly by excessive water retention. This leads to interference with aerobic adenosine triphosphate (ATP) production, mitochondrial mediated apoptosis, and eventually the destruction of the plasma membrane as the hepatocytes lyse from the excessive hydration [16,25]. The transaminases are released from the cytoplasm and become detectable by way of testing for ALT and AST in blood serum.

Our histopathological examination also showed staining that was consistent with glycogen depletion at high levels of BPA exposure. The glycogen depletion may be a symptom of only hepatic trauma [30], it may be a consequence of neuropeptide upregulation, or it may be a combination of both. From the available data, we can report the glycogen depletion, but can only speculate about what may be causing it.

It is quite possible that BPA may damage hepatocytes in a similar way to that of the toxins mentioned above. Our results have shown significantly increased ALT and AST levels when compared to the control group at both low dose and high dose exposure. In the high dose BPA group, the AST and ALT levels were nearly double that of the control group. Other studies have shown increased ALT levels in rat models after moderate duration BPA exposure [16]. Since the potential dangers of BPA exposure have only come to light within the last ten years, there is a limited amount of literature with regards to the effects of BPA on hepatocytes and hepatic tissue in general. However, some recent studies have shown that BPA exposure caused hepatic trauma by way of oxidative stress as well as mitochondria-mediated apoptosis [16,33]. Other studies have shown that high and low dose BPA exposure caused genomic damage as well as alterations in liver enzyme levels [15,17]. All of the in vivo studies were carried out using rat models. To the best of our knowledge, our study is the first in vivo study to use an animal model (porcine) that more closely approximates human physiology.

Although the BPA exposed liver samples in our study showed no changes in the hepatic lobular structure, they did show observable levels of vacuolar degeneration, sinusoidal dilatation, and vascular congestion. At high BPA exposure levels, vacuolar degeneration was also observed. These findings are consistent with the literature cited above. Therefore, it is reasonable to hypothesize that BPA exposure in developing children may also be causing similar hepatic trauma depending on the level of exposure. Our results have shown observable hepatic injury in a large animal model that approximates human development after only 28 days of BPA exposure. The histopathology presented in the figures taken together with the increased ALT and AST levels, plus the previous literature based on earlier rodent models, is highly suggestive of BPA inducing hepatocyte damage after only short-term exposure at currently suggested safe-levels. The mechanism of how this may be happening is currently not well-understood. Since the first major organ that ingested materials come into contact with is the liver, BPA could be causing liver damage in developing children who are exposed to this common endocrine disruptor, but future research is needed.

Knowledge of the anatomy and physiology of the intrinsic innervation of hepatic tissue is relatively recent, with the first detailed articles concerning hepatic intrinsic nerves published within the last thirty-five years [18,34,35]. More recently, neural markers have been used to study hepatic innervation by immunofluorescence. These studies are often specialized into examining the nerve-fibers of the sympathetic nervous system or the parasympathetic nervous system. For the parasympathetic nervous system (PSNS) markers such as acetylcholinesterase (AChE) and VAChT are most often used [19,36]. This study made use of VAChT in order to specifically investigate the hepatic PSNS. Although there are several studies which have investigated neural “gut-brain” markers, to the best of our knowledge, this is the first study to investigate changes in hepatic neural markers after BPA exposure.

VAChT is an enzyme important for the proper neural functioning of the PSNS (parasympathetic nervous system). The PSNS is the most commonly used part of the autonomic nervous system (ANS), and is often known as the “rest and digest” portion of the ANS [37,38]. The PSNS is characterized by its use of acetylcholine as the main neurotransmitter for cellular communication. Tissues associated with the PSNS are characterized as having many acetylcholine receptors [18,37,38]. The results of this study found no significant changes in the VAChT^+^ nerve fibers. Therefore, BPA exposure in juvenile porcine models does not lead to an upregulation of the hepatic PSNS in a manner which can be detected with statistical significance. This does not mean that the PSNS is not upregulated at all. It may be upregulated, but not at a significantly detectable level. What may be of more interest is the fact that some of the neuronal peptides colocalized with VAChT did show significant upregulation.

VAChT has quite often been visualized by way of immunohistochemistry using porcine animal models. For example, the team of Gonkowski used VAChT to study the PSNS of the porcine ileum [38], while the team of Wojtkiewicz used VAChT to study the PSNS of the porcine esophagus [39]. The aforementioned articles used immunostaining techniques very similar to this study. This is relevant since the liver is the first major organ to come into contact with what is absorbed by the digestive system. These “gut-brain” peptides are an important field of study when looking at dietary environmental exposure [40]. Although the hepatic PSNS, through the visualization of VAChT, did not show significant increases, it is possible that the other half of the autonomic nervous system (the sympathetic nervous system) may be showing significant upregulation by itself or colocalized with neuronal “gut-brain” markers. In this study, there were neuronal markers that showed statistically significant increased upregulation.

In previous studies, markers such as CGRP, SP, GAL, PACAP, and CART have been used to observe nerve-fibers, quite often in the gastrointestinal system. CGRP has been used as a spinal afferent marker [41] and has a role in cerebrovascular regulation [42], while substance P has been used as a similar marker, but more extensively [43]. SP has a role in inflammatory diseases, nociception, and depression [44,45]. Galanin has been found to potentiate the effects of norepinephrine, is a neuromodulator affecting glucose production in the liver [46], and has been correlated with diabetes mellitus in children [47,48]. Unfortunately, the mechanisms are not well understood. PACAP has been linked with food intake, appetite control, glucose metabolism [49,50], and more recently with psychological and behavioral disorders such as post-traumatic stress disorder (PTSD), hyperactivity, memory impairment, and stress-related illnesses [51,52,53]. In a similar way to the early studies of PACAP, CART has been linked with food intake, appetite control, and the regulation of lipids in adipose tissue [54,55,56]. More recently, abnormal patterns of CART have been associated with diabetes mellitus [57]. The mechanisms are not fully understood, but appear to be highly complex with multiple gene involvement [55].

Of the nerve fibers tested, only VAChT colocalized with GAL showed significant upregulation after bisphenol exposure at currently accepted recommended safe exposure levels. Incorrect expression of GAL has been indicated in problems with energy metabolism in children, including diabetes mellitus [46,47,48]. Again, the mechanisms for these correlations are not well understood. The results above show elevated VAChT^+^/GAL^+^ nerve fibers at recommended safe levels, when compared to that of control samples. Furthermore, it is a rather dramatic increase for a level that is considered to be safe. Moreover, at ten times the recommended safe-level, which is not out of the bounds of possibility due to the ubiquitous nature of bisphenol in our modern environment, the upregulation of GAL^+^/VAChT^+^ was very much higher than that of the control samples. These increases in GAL^+^/VAChT^+^ nerve fibers could be a contributing factor to early onset diabetes and obesity in children and young adults who are exposed to BPA if the porcine models are an accurate depiction of human development, regardless of whether the dosage is legally acceptable or not. However, further research is needed to validate the above hypothesis.

At ten times the legally accepted safe level of bisphenol, three colocalized neuronal markers showed significant upregulation. These three were the VAChT^+^/GAL^+^ (previously discussed), VAChT^+^/CART^+^, and VAChT^+^/CGRP^+^ nerve fibers. At an exposure of ten times higher than recommended safe levels, the VAChT^+^/CART^+^ immunoreactive nerve fibers were nearly two times higher than that of the control samples. Since CART has been shown to control metabolism via the CNS, it has a key role in the proper functioning of appetite regulation [40,54,55,56,57,58,59]. Therefore, increased CART markers within the PSNS could be a contributing factor to increased obesity in children and young adults. This could be a factor in the correlation between CART and diabetes mellitus [57] since the liver is responsible for the homeostasis of glucose levels, but further research would need to be performed to verify such a claim. Childhood obesity and BPA exposure has been correlated [60], and a cross-sectional study in Chinese school children correlated BPA exposure with increased body mass index (BMI) [61]. Both studies showed that there was a statistically significant correlation between urinary BPA concentrations and increased BMI as well as obesity in children and adolescents. At a bisphenol exposure of ten times higher than recommended safe levels, the upregulation of VAChT^+^/CGRP^+^ nerve fibers was significantly higher than that of the control samples. CGRP is a potent vasodilator and has been linked with metabolic regulation [41,42]. Increased upregulation may contribute to childhood obesity and diabetes using the same neuromodulating mechanisms as those described in VAChT^+^/CART^+^ nerve fiber signaling [40,54,55,56,57,58,59,61]. The currently available mechanisms linking changes in these markers with childhood obesity and diabetes are described below.

The three neural peptides mentioned above (CART, CGRP, and GAL) have been correlated with childhood obesity and diabetes in several studies, previously cited. Although few mechanistic studies have been published yet, a recent study found that CART modulated mesolimbic dopamine systems and affected reward and reinforcing behaviors which were “linked to eating disorders, including obesity and anorexia” [62]. Furthermore, CART, CGRP, and GAL have been more recently described as gut-brain markers as well as being present in the central nervous system [63]. Other studies have shown these gut-brain markers to be linked with diabetes and obesity in children mainly through altered regulation of metabolic gene expression [64,65,66,67]. Unfortunately, detailed mechanisms are an area of future research and are not available at this time.

Although this study did not notice any statistically significant changes in SP or PACAP immunoreactive nerve fibers, both of those neuronal markers were found to increase. BPA exposure has been correlated with hyperactivity, anxiety, and other behavioral problems in children [68,69,70,71,72]. A recent study showed that increased neuronal expression of several dozen genes during the early post-natal period was correlated with altered levels of anxiety [73]. The mechanisms appear to be highly complex, however, and only correlation studies are currently available. Furthermore, a previous study correlated increased PACAP expression with post-traumatic stress disorder [51]. Therefore, it may be valid in future research to investigate a link between BPA exposure, neural upregulation, and psychological disorders in children. However, these markers were not statistically significant in this study, and no significant conclusions can be drawn at the current time.

The histopathological examination of the liver showed changes consistent with glycogen depletion at high BPA exposure levels. It is possible that the increased GAL, CART, and CGRP could be causing an altered metabolism, which is evident in decreased hepatic glycogen levels. This explanation could tie together the histopathological observations with the upregulation of these neurochemical peptides. This, taken together with the literature cited above, could become a stepping off point to investigate the mechanisms of how BPA is altering hepatic metabolism in developing organisms. On the other hand, the glycogen depletion could simply be another indicator of hepatic trauma with no neurochemical involvement. Further research should be performed to investigate the phenomenon.

## 4. Materials and Methods

### 4.1. The Animal Models Used in This Study

The present study was completed on fifteen immature sows of the Piétrain x Duroc breed at the age of 8 weeks and about 18–20 kg body weight. Pigs were kept under typical laboratory conditions adapted for this animal species. The experiment was performed in compliance with the instructions of the Local Ethical Committee for Experiments on Animals in Olsztyn (Poland) decision number (28/2013; 22.05.2013).

After a 3-day adaptive period, the pigs were randomly divided into three experimental groups: (1) control group-placebo (empty gelatin capsules for 28 days during feeding); (2) experimental group I (received BPA capsules at a dose acceptable under European legislation—0.05 mg (50 μg) /kg bw/day); (3) Experimental group II (received BPA capsules at a dose 10 times higher than the acceptable level—0.5 mg /kg bw/day). Every four days before the morning feeding, all animals were weighed in order to determine their body weight and calculate the proper dosage of BPA.

### 4.2. Tissue Collection, Fixation, and Immunolabeling

After 28 days of BPA administration, the animals were premedicated with Stressnil (Janssen, Belgium, 75 μL/kg of body weight, intramuscular). After about 30 min, the animals were euthanized using an overdose of sodium thiopental (Thiopental, Sandoz, Kundl-Rakúsko, Austria, intravenous). Tissues were collected from all sows. Sections of liver tissues were fixed in 4% buffered paraformaldehyde, rinsed in phosphate buffer for three days, and kept in 18% sucrose at 4 °C. After at least two weeks, the fragments of liver were frozen at −23 °C and cut into 10 μm-thick sections using a microtome (Microm, HM 525, Walldorf, Germany). The sections were subjected to a routine double-labeling immunofluorescence technique according to the method described previously by Gonkowski [38,40] and Wojtkiewicz [74,75]. A condensed description of the method is as follows: 45 min of drying; incubation with a blocking solution, which included 10% normal goat serum, 0.1% bovine serum albumin, 0.01% NaN_3_, Triton X−100, and thimerozal in phosphate buffered saline (PBS) for 1 h; overnight incubation with a mixture of two “primary” antibodies raised in different species and directed towards vesicular acetylcholine transporter (VAChT), and one of the aforementioned substances i.e., CART, substance P, CGRP, GAL, or PACAP; incubation (for 1 h) with species-specific antisera conjugated to Fluorescein (FITC) or biotin, which was visualized by a streptavidin-CY3 complex (the specification of intravenous primary and secondary antibodies used in the present study is shown in Table 2). Rinsing with PBS (3 × 10 min, pH 7.4) was performed between each of the stages.

During the present investigation, the standard controls of the specificity of “primary” antibodies were performed. These included pre-absorption of the particular antisera with appropriate antigens, as well as “omission” and “replacement” tests that completely eliminated immunofluorescence signals.

### 4.3. The Histopathological Investigation

To evaluate the number of VAChT^+^ intrahepatic nerves, the nerves were counted using a microscopic observation field (0.1 mm^2^). Nerves immunoreactive to VAChT were counted in four sections of the liver per animal (in five randomly selected observation fields per section) and the obtained data were pooled and presented as a mean ± SEM. The method used to evaluate the neurochemical characteristics of VACHT^+^ nerves consisted of determining what percentage of all such nerve fibers were simultaneously immunoreactive to each of the other substances included in the investigation. To this end, at least 300 VAChT-labeled nerves in each studied animal were examined for immunoreactivity to the other particular substance studied. VAChT-positive nerve fibers were considered as representing 100%. The evaluation of immunopositive nerve fibers and the counting of nerves were performed by two independent investigators. Double-labeled nerve fibers were visualized under an Olympus BX51 microscope equipped with epi-fluorescence and appropriate filter sets. The obtained results were pooled and presented as a mean ± SEM. To prevent double counting of the same nerves, the sections of liver evaluated during the present study were located at least 100 µm apart. Statistical analysis was carried out via Student’s *t* test (Graphpad Prism v. 6.0; GraphPad Software Inc., San Diego, CA, USA). The differences were considered statistically significant at *p* ≤ 0.05.

The histopathological investigation of the porcine hepatic tissue was performed using the following procedure: tissue samples were fixed in buffered 10% formalin. Subsequently, they were dehydrated in graded ethanol and embedded paraffin. Four-micrometer (4 μm) thick sections were placed upon silanized slides, deparaffinized, rehydrated in graded ethanol, and then stained with hematoxylin and eosin.

## 5. Conclusions

Although the BPA exposed liver samples showed no changes in the hepatic lobular structure, they did show observable levels of vacuolar degeneration, sinusoidal dilatation, and vascular congestion. These are indicative of early hepatic injury, especially after exposure to toxic substances. Other similar studies have been performed in vivo on rodents, and they have also observed hepatic trauma after both high and low dose BPA exposure. This is the first study to our knowledge examining the effects of BPA on porcine liver, which is a closer approximation of human physiology than mice or rat. If these trends are extrapolated to humans, then children continually exposed to bisphenol compounds for several years during their childhood could be at a significantly higher risk of liver damage as well as altered metabolism. To the best of our knowledge, damage to hepatocytes has not been correlated with upregulation of the immunoreactive nerve fibers used here. From the literature, we cannot support an argument that altered patterns of innervation cause damage to hepatocytes. Therefore, it is our opinion that in this study, BPA most likely caused early signs of hepatocyte damage independently of altering neurochemical patterns. Furthermore, hepatic glycogen depletion was observed, but it is not clear whether this was due to the upregulation of nerve-fibers in this study or if it was a product of hepatic damage. Of course, future research of the mechanisms involved is highly recommended.

It is not unreasonable to assume that a developing child could be exposed to ten times the suggested safe limit of bisphenol A. This EDC can be found everywhere today and many parents are still not aware of the dangers of BPA exposure [1,2,3,4,5]. Therefore, it may be recommended to revise the suggested safe levels of BPA exposure downward, as well as increase public awareness of the potential dangers of BPA—especially with regards to children. Moreover, outside of the PSNS, bisphenol A may be causing undesirable effects in children and young adults.

## Figures and Tables

**Table 1:** Neurochemical characteristics and changes in neuropeptide expression in intrahepatic sympathetic nerves under physiological conditions and after bisphenol A administration. The studied groups of animals were: CTRL—control. E1—experimental group 1 (low dose of bisphenol A), E2—experimental group 2 (high dose of bisphenol A).

Neurochemical Characteristic	Groups of Animals		
	CTRL	E1	E2
VAChT^+^	27.4 ± 4.41 ^b^	29.8 ± 3.28	34.8 ± 4.25 ^b^
VAChT^+^/CART^+^	10.6 ± 2.80 ^a,b^	15.4 ± 2.25 ^a,c^	20.8 ± 2.52 ^b,c^
VAChT^+^/SP^+^	6.2 ± 1.93 ^b^	8.8 ± 1.77	8.9 ± 1.41
VAChT^+^/CGRP^+^	12.0 ± 3.11 ^b^	13.6 ± 2.86 ^c^	20.4 ± 4.43 ^b,c^
VAChT^+^/GAL^+^	3.4 ± 1.33 ^a,b^	9.1 ± 2.51 ^a^	12.2 ± 3.02 ^b^
VAChT^+^/PACAP^+^	11.8 ± 3.25 ^a,b^	17.6 ± 4.23 ^a^	19.4 ± 5.81 ^b^

^a^: indicates differences for particular substances between CRTL and E1; ^b^: indicates differences for particular substances between CRTL and E2; ^c^: indicates differences for particular substances between E1 and E2. VAChT: vesicular acetylcholine transporter; CART: cocaine and amphetamine regulated transcript; SP: substance P; CGRP: calcitonin gene regulated peptide; GAL: galanin; PACAP: pituitary adenylate cyclase activating polypeptide.

**Table 2:** List of primary and secondary antibodies used in this study.

Primary Antibodies				
Antisera	Code	Host Species	Dilution	Supplier
**VAChT**	H-V007	Goat	1:2000	Phoenix Europe; www.phoenixpeptide.com
**CART**	H-003-61	rabbit	1:22,000	Phoenix Europe; www.phoenixpeptide.com
**SP**	8450-0505	rabbit	1:10,000	Biogenesis Inc.; www.biogenesis.co.uk
**CGRP**	11189	rabbit	1:10,000	MP Biomedicals; www.mpbio.com
**GAL**	RIN7153	rabbit	1:10,000	Peninsula Labs, US; see Bachem AG; www.bachem.com
**PACAP**	IHC 8922	rabbit	1:20,000	Bachem AG; www.bachem.com
**Secondary Antibodies**				
**Reagent**			**Dilution**	**Supplier**
Donkey anti-goat IgG (H + L) conjugated with FITC			1:800	715-095-003; Jackson IR Lab, US; www.jacksonimmuno.com
Biotinylated goat anti-rabbit immunoglobulins			1:1000	E0432, DAKO Corporation, US; www.dakousa.com
Biotin conjugated F(ab)’ fragment of affinity Purified anti-rabbit IgG (H + L)			1:1000	711-1622, BioTrend, Germany; www.biotrend.com
CY3-conjugated Streptavidin			1:9000	016-160-084, Jackson IR Lab, US; www.jacksonimmuno.com

## Abbreviations

AChE	acetylcholinesterase
ALT	alanine aminotransferase
AST	aspartate aminotransferase
ANS	autonomic nervous system
BPA	bisphenol A
CART	cocaine and amphetamine regulated transcript
CGRP	calcitonin gene regulated peptide
EDC	endocrine disrupting compound
GAL	galanin
PACAP	pituitary adenylate cyclase activating polypeptide
PSNS	parasympathetic nervous system
SNS	sympathetic nervous system
SP	substance P
VAChT	vesicular acetylcholine transporter
